# Genetic and Physiological Characterization of Two Clusters of Quantitative Trait Loci Associated With Seed Dormancy and Plant Height in Rice

**DOI:** 10.1534/g3.112.005041

**Published:** 2013-02-01

**Authors:** Heng Ye, Donn H. Beighley, Jiuhuan Feng, Xing-You Gu

**Affiliations:** * Plant Science Department, South Dakota State University, Brookings, South Dakota 57007; † Department of Agriculture, Southeast Missouri State University, Malden, Missouri 63863

**Keywords:** seed dormancy, plant height, quantitative trait locus, gibberellin signaling, weedy rice

## Abstract

Seed dormancy and plant height have been well-studied in plant genetics, but their relatedness and shared regulatory mechanisms in natural variants remain unclear. The introgression of chromosomal segments from weedy into cultivated rice (*Oryza sativa*) prompted the detection of two clusters (*qSD1-2/qPH1* and *qSD7-2/qPH7*) of quantitative trait loci both associated with seed dormancy and plant height. Together, these two clusters accounted for >96% of the variances for plant height and ~71% of the variances for germination rate in an isogenic background across two environments. On the initial introgression segments, *qSD1-2/qPH1* was dissected genetically from *OsVp1* for vivipary and *qSD7-2/qPH7* separated from *Sdr4* for seed dormancy. The narrowed *qSD1-2/qPH1* region encompasses the *semidwarf1* (*sd1*) locus for gibberellin (GA) biosynthesis. The *qSD1-2/qPH1* allele from the cultivar reduced germination and stem elongation and the mutant effects were recovered by exogenous GA, suggesting that *sd1* is a candidate gene of the cluster. In contrast, the effect-reducing allele at *qSD7-2/qPH7* was derived from the weedy line; this allele was GA-insensitive and blocked GA responses of *qSD1-2/qPH1*, including the transcription of a GA-inducible α-amylase gene in imbibed endosperm, suggesting that *qSD7-2/qPH7* may work downstream from *qSD1-2/qPH1* in GA signaling. Thus, this research established the seed dormancy-plant height association that is likely mediated by GA biosynthesis and signaling pathways in natural populations. The detected association contributed to weed mimicry for the plant stature in the agro-ecosystem dominated by semidwarf cultivars and revealed the potential benefit of semidwarf genes in resistance to preharvest sprouting.

Seed dormancy and plant height contribute to adaptation by regulating the timing of germination and plant architecture, respectively. Both traits have been intensively selected during domestication or breeding, resulted in the divergence between cereal crops and their wild relatives. For example, domestication tended to reduce seed dormancy to synchronize germination, which also caused the problem of preharvest sprouting in production (Harlan *et al.* 1973; Bewley and Black 1994); crop breeding has been in favor of semidwarf varieties to prevent lodging since the “Green Revolution” commenced in the middle of the last century (Khush 2001). Despite years of detached research on seed dormancy and plant height, little attention has been paid to their relatedness and shared regulatory mechanisms. Thus, it remains unclear whether and how the selection for one of the two traits impacted the other.

Many seed dormancy and plant height genes or quantitative trait loci (QTL) were identified, but none of them is known to influence both of the two traits. In wheat (*Triticum aestivum*), the *Reduced height1* genes encode DELLA proteins, which act as master regulators of gibberellin (GA) signaling, and the gain-of-function mutants *Rht-B1b* and *Rht-D1b* produce truncated DELLA proteins resulting in reduced GA response and plant height (Peng *et al.* 1999). In rice (*Oryza sativa*), the *Semi-dwarf1* gene (*Sd1* or *GA20ox2*) encodes GA20-OXIDASE2 catalyzing the second-to-last step of the GA biosynthesis, and the *sd1* loss-of-function mutants produce defective enzymes resulting in reduced GA and plant height (Ashikari *et al.* 2002; Monna *et al.* 2002; Spielmeyer *et al.* 2002). These and other genes for GA biosynthesis or signaling were not reported to have an effect on germination. On the other hand, the seed dormancy QTL *DOG1* in Arabidopsis, and *Sdr4* and *qSD7-1* in rice were map-based cloned. *DOG1* and *Sdr4* encode predicted proteins with unknown molecular functions (Bentsink *et al.* 2006; Sugimoto *et al.* 2010). The *qSD7-1* underlying gene encodes a basic helix-loop-helix family transcription factor that enhanced the biosynthesis of the dormancy-inducing hormone abscisic acid in developing seeds (Gu *et al.* 2011). None of the dormancy genes was reported for an effect on plant height. Thus, characterization of additional QTL for seed dormancy may help clarify the relationship between the two adaptive traits.

Weedy rice, the *Oryza* genus plants adapted to rice-growing areas, retained some wild traits (*e.g.*, strong seed dormancy) and also developed crop mimicries for agronomic traits such as plant height (Oka 1988; Delouche *et al.* 2007). The previous research identified several QTL for seed dormancy (*qSD*) in a primary segregating population (BC_1_F_1_) developed from the cross between a weedy and a cultivated line of rice (Gu *et al.* 2004). In the subsequent research to transfer individual QTL alleles from the weedy into the cultivated rice, *qSD1-2* and *qSD7-2* were also associated with plant height, or co-located with plant height QTL in different advanced generations. Interestingly, the isolated *qSD1-2* region encompasses the loci *GA20ox2* and *OsVp1* (ortholog of the Maize *Viviparous1* gene) (McCarty *et al.* 1991) and *qSD7-2* was colocated with the *Sdr4* seed dormancy QTL (Lin *et al.* 1998; Gu *et al.* 2004). Therefore, the objectives of this research were: (1) to estimate genetic component effects of the *qSD1-2* and *qSD7-2* regions on both seed dormancy and plant height in an isogenic background; (2) to determine allelic relations between *qSD1-2* and *GA20ox2* or *OsVp1*, and between *qSD7-2* and *Sdr4*; and (3) to characterize the QTL clustered in the *qSD1-2* and *qSD7-2* regions for GA responses, including germination, seedling elongation, and transcription of a GA-induced *α*-amylase gene in the endosperm of imbibed seeds.

## Materials and Methods

### Plant materials and breeding scheme

**Figure 1:**
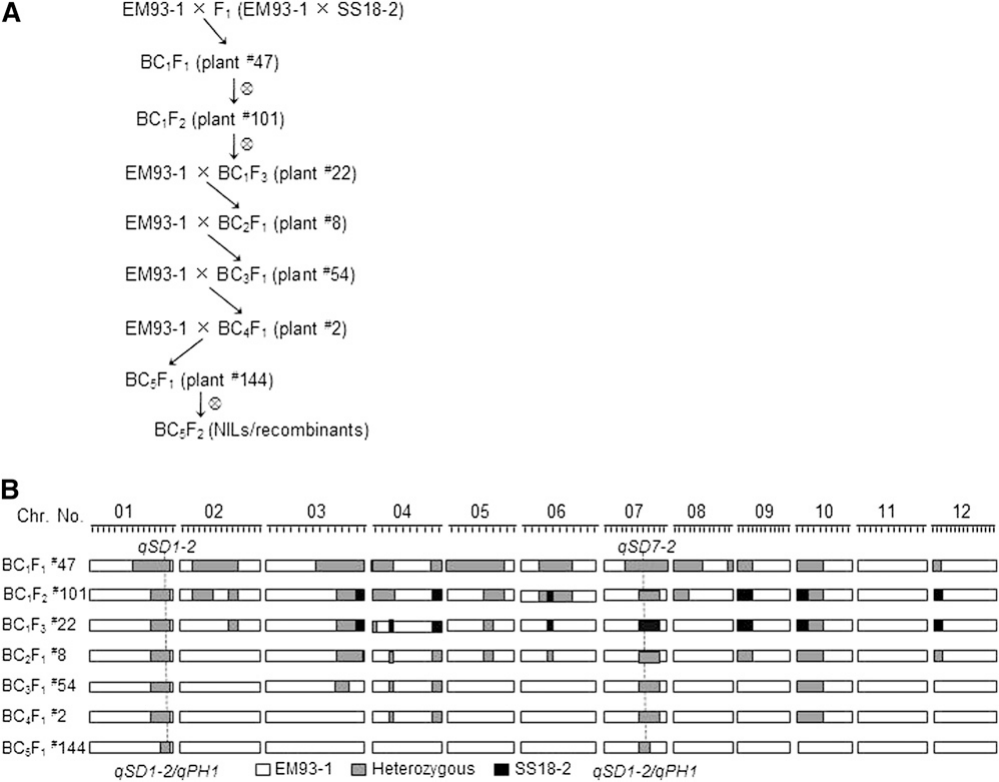
Introgression of chromosomal segments containing seed dormancy QTL. (A) Breeding scheme. Recurrent backcross combined with a marker-assisted selection was used to introduce chromosomal segments from the weedy rice line SS18-2 into the genetic background of the cultivated rice line EM93-1. Plants in parentheses were selected based on marker genotypes to develop the next generation of segregating population or NILs. (B) Graphic representation of genotypes for the selected plants. Tick markers on chromosomes 01−12 indicate marker positions mapped based on the BC_1_F_1_ population (Gu *et al.* 2004). Horizontal bars indicate that the plant was homozygous for an EM93-1 (empty)- or SS18-2 (dark)-derived segment, or heterozygous (gray). Vertical lines position the seed dormancy/plant height QTL clusters *qSD1-2/qPH1* and *qSD7-2/qPH7*.

The *qSD7-2* QTL was identified from the BC_1_F_1_ (EM93-1//EM93-1/SS18-2) population and mapped on the long arm of chromosome 7 (Gu *et al.* 2004), whereas the *qSD1-2* was detected in two BC_1_F_1_ plant-derived BC_1_F_2_ populations and located on the long arm of chromosome 1 (Ye *et al.* 2010). SS18-2 is a weedy (*O. sativa*) and EM93-1 is a cultivated line of rice, and both lines are semidwarf in plant height. The parental and hybrid F_1_ (EM93-1/SS18-2) generations and the BC_1_F_1_, BC_1_F_2_, and BC_1_F_3_ populations (Figure 1A) were used to infer an inheritance pattern and to map QTL for plant height.

Recurrent backcrossing, combined with marker-assisted foreground and background selection, started with a BC_1_F_3_ plant and continued for four (BC_2_F_1_−BC_5_F_1_) generations to transfer only the *qSD1-2*- and *qSD7-2*-containing segments from SS18-2 into the EM93-1 background (Figure 1). The BC_5_F_1_ heterozygote for these two segments (*e.g.*, plant ^#^144 in Figure 1B) was advanced to the BC_5_F_2_ and higher generations to select a digenic system of all nine digenic genotypes, nearly isogenic lines (NILs) for the four homozygotes, and recombinants for each of the two QTL regions.

### Marker genotyping and genotypic selection

Rice microsatellite (RM) markers on the framework linkage map of weedy rice (Gu *et al.* 2004) were used to genotype advanced backcross populations for QTL mapping. Markers flanking the *qSD1-2* and *qSD7-2* peak positions were used to genotype the BC_5_F_2_s and their progeny lines to select for the digenic system, NILs, and recombinants. The digenic system consisted of all nine digenic genotypes for the two QTL and the selected genotypes were similar in frequency to minimize biased estimates of genetic component effects. The NILs were progeny lines of the four homozygotes from the digenic system and were designated 1^−^1^−^7^−^7^−^, 1^+^1^+^7^−^7^−^, 1^−^1^−^7^+^7^+^, and 1^+^1^+^7^+^7^+^, with the superscripts indicating germination/height-promoting (+) or -reducing (−) alleles at *qSD1-2* on chromosome 1 or *qSD7-2* on chromosome 7. The recombinants were identified from about 500 plants segregating for one of the two initial introgression segments. Marker genotyping was conducted on 3- to 4-wk-old seedlings using the previously described methods (Gu *et al.* 2004).

On the initial introgression segments from SS18-2, the *qSD1-2* interval encompasses the loci *sd1* (*Os01g66100*) and *OsVp1* (*Os01g68370*), and the *qSD7-2* interval covers the locus *Sdr4* (*Os07g39700*), according to the reference genome sequence of japonica rice [(IRGSP 2005) Gramene Database release 35 at http://www.gramene.org]. Thus, more than 20 RM markers physically aligned to the two QTL regions on the reference genome (McCouch *et al.* 2002) were screened for polymorphism between EM93-1 and SS18-2. Polymorphic markers were used to delimit the SS18-2−derived segments retained in individual recombinants. In addition, the marker *sd1*(k), which targets the 383-bp functional deletion within *sd1* (Monna *et al.* 2002), was used to genotype the parental and recombinant plants to infer allelic differentiation at the GA synthesis gene. Based on the marker genotypes, six recombinants that were heterozygous for different sub-segments of the *qSD1-2*- or *qSD7-2*-containing regions were selected for progeny testing. About 90 seedlings from each of the progeny lines were genotyped with a marker on the heterozygous region and grown in the greenhouse for phenotypic assessment.

### Greenhouse and field experiments

Genetic component effects of the two QTL-containing regions on seed dormancy and plant height were evaluated in a greenhouse and a field experiment. Fully after-ripened BC_5_F_2_ seeds were germinated in a 30° incubator for 5 d, and sprouting seeds transferred to 200-cell Plug Trays filled with rice nutrient solution (Yoshida *et al.* 1976) for 4 wk to synchronize seedling size. For the greenhouse experiment, ~24 seedlings selected for each of the nine digenic genotypes were transplanted into pots (one plant/pot), which were filled with a mixture of clay soil and potting medium (Sunshine Mix #1; SUNGRO Horticulture Ltd., Canada) and placed in watertight containers (60 cm × 120 cm). The day/night temperatures in the greenhouse were set at 29/21°, and photoperiods were natural for the summer or 12 hr for the winter season. Plants were tagged for flowering date when the first panicle in a plant emerged from the leaf sheath. Seeds were harvested at 40 d after flowering and air-dried in the greenhouse for 3 d before storage in a −20° freezer to maintain the dormant status. For the field experiment, ~25 seedlings for each of the nine genotypes were transplanted in individual plots, at a density of 16 cm by 30 cm, on the Rice Research Farm of Southeast Missouri State University in Malden, MO. Seed harvesting and storage were conducted using the aforedescribed methods.

### Phenotypic assessment for seed dormancy and plant height

Seed dormancy was quantified by germination rate under controlled conditions. Before germination, seed samples from individual plants were left at the room temperature (~24°, *i.e.*, after-ripening) for varying lengths (0−30 d depending on experiments) to maximize the variance in a population or to estimate the after-ripening period required for a genotype to achieve 50% germination. A germination experiment consisted of three replications for all plants from a segregating population or a progeny line. A replicate of approximately 30 seeds distributed in 9-cm Petri dishes, each lined with a filter paper and wetted with 8 mL of deionized water, was germinated at 30°, 100% relative humidity and in dark for 7 d. Germinated seeds (protrusion of the radicle from the hull by ≥3 mm) were counted daily from 2 to 7 d. Germination percentage (*x*) for a sample was transformed by sin^−1^(*x*^−0.5^) to improve the normality of the frequency distribution.

Plant height was measured as the length of the main stem from the soil surface to the top of the panicle at maturation and recorded for single plants cultivated using the aforedescribed methods, with the exception of the parental, F_1_ and BC_1_F_1_ generations. Five plants from each of the parental and F_1_ generations and 156 BC_1_F_1_ plants were duplicated four times by a splitting-tiller technique at the seedling stage; the genetically identical duplicates were grown in large pots (28 cm diameter and 25 cm height) placed in the same greenhouse (Gu *et al.* 2004). For these generations, plant height data recorded for the four tiller-derived plants were averaged for genetic and QTL analyses.

### QTL mapping and genetic effect estimation

Linkage maps constructed based on the BC_1_F_1_, BC_1_F_2_, and BC_1_F_3_ populations (Gu *et al.* 2004, Ye *et al.* 2010) were used to scan for plant height QTL. The map construction and QTL mapping were conducted using the backcross F_1_ model for the BC_1_F_1_ data, or using the intercross F_2_ model for the BC_1_F_2_ and BC_1_F_3_ data. The composite interval mapping program of Windows QTL Cartographer v2.5 (Wang *et al.* 2006) was used to generate likelihood ratio distributions along all (BC_1_F_1_) or part (BC_1_F_2_ and BC_1_F_3_) of the 12 chromosomes to infer QTL segregating in the populations. Parameters set to run the program included Kosambi’s map function, 1-cM walking speed, and 1000 random permutations at a type-I error rate of 5%.

Genetic component effects of the narrowed *qSD1-2* and *qSD7-2* regions on seed dormancy and plant height were estimated using the additive (*a*)-dominance (*d*)-epistasis (*I*) model for two loci,

(Model 1) $$y_{ijk}=\mu +a_{1}x_{1}+d_{1}z_{1}+a_{2}x_{2}+d_{2}z_{2}+I_{aa}w_{aa}+I_{ad}w_{ad}+I_{da}w_{da}+I_{dd}w_{dd}+\varepsilon_{ijk}$$

where, *y_ijk_* is the phenotypic value for the *k*th plant (*k* = 1 to N, the population size) of a digenic genotype for *qSD1-2* (*i*) and *qSD7-2* (*j*), with *i* and *j* = 0, 1, or 2 representing the copy number of the SS18-2-derived allele; *µ* is the model mean; *x_1_* and *x_2_* are variables for additive (linear) components of loci *i* and *j*, respectively, with *x* coded as –1, 0, and 1, when *i* or *j* = 0, 1, and 2, respectively; *z_1_* and *z_2_* are the variables for the dominance (quadratic) components of loci *i* and *j*, respectively, with *z* coded as 1 and 0 when *i* or *j* = 1 and 0 or 2, respectively; *w_aa_*, *w_ad_*, *w_da_*, and *w_dd_* are the variables for additive-additive (*w_aa_*), additive-dominance (*w_ad_*) or dominance-additive (*w_da_*), and dominance-dominance (*w_dd_*) types of epistasis between the two loci, with each type coded as the product of codes for the corresponding additive or dominance variables; *a_1_*, *d_1_*, *a_2_*, *d_2_*, *I_aa_*, *I_ad_*, *I_da_*, and *I_dd_* are partial regression coefficients for the corresponding variables that estimate component *a*, *d*, and *I* effects; and *ε_ijk_* is the residual including random error and environmental effects. Regression analysis for the greenhouse and field data sets was implemented separately using the SAS REG procedure, with a stepwise selection at a significance level of 5% (SAS Institute 1999).

Data from the six progeny lines were analyzed by linear correlation to infer if the recombinants differentiate at *qSD1-2*, or *qSD7-2*, or known gene (*sd1*, *OsVp1*, or *Sdr4*). Marker genotypes like EM93-1, heterozygous, and like SS18-2 in a progeny line were coded as 0, 1, and 2, respectively, to correlate with the trait values for germination and plant height. A significant marker-trait correlation indicates that the recombinants retained a copy of functional dormancy/plant height gene on the marked heterozygous region. A nonsignificant correlation suggests that the recombinant is fixed for the QTL/gene or that the QTL underlying gene is located outside the marked heterozygous region. For the progeny lines with a significant marker-trait correlation, the data were also used to estimate additive and dominance effects of the QTL/gene using the model,

(Model2) $$y_{ik}=\mu +ax+dz+\varepsilon_{ik}$$

Model (2) was modified from model (1) by removing variables related to the second locus.

### GA induced seedling growth, germination, and gene expression experiments

The four NILs were used to evaluate genotypic responses to exogenous GA (GA_3_; Acros Organic, NJ) for germination, seedling growth, and the expression of the GA-inducible *α*-amylase gene *Amy1A* to infer relations between the seed dormancy/plant height QTL and GA synthesis or signal transduction. To evaluate the germination response, dried seeds without an after-ripening treatment were soaked in each of the 0 (control), 0.1, 1.0, 10.0, and 100.0 µM GA_3_ solutions and incubated under the aforedescribed conditions for 7 d. The sample size was about 40 seeds, and the germination experiment was replicated three times for 10 plants from each of the four NILs.

Two experiments were conducted to evaluate GA effects on seedling growth. In the initial experiment, 10 seedlings (2-d-old) from an NIL were grown in a 9-cm Petri dish, which was lined with two layers of filter paper and irrigated with one of the 0 (control), 0.1, 1.0, 10.0, and 100.0 µM GA_3_ solutions, in a growth chamber set for 30° and 16-hr light. Seedling length was measured after 7 d. In the second experiment, 18 seedlings at 5 d of age were planted in nutrient solutions (Yoshida *et al.* 1976) with different GA_3_ concentrations in the greenhouse. The length of the second foliage leaf sheath was measured every 3 d during the following 15-d period.

The expression of *Amy1A* in the aleuron layer was induced by GA from the embryo to synthesize *α*-AMYLASE1A required to hydrolyze starchy endosperm in imbibed seeds of rice (Kaneko *et al.* 2002). Quantitative real-time polymerase chain reaction (PCR) assays were used to compare genotypic differences in the transcription level of *Amy1A*. Three samples of 50 seeds from an RIL were incubated in the 0 (control) or 10 µM GA_3_ solution for 36 hr. Total RNA was extracted from the endosperm tissue cut from the imbibed seeds using TRI Reagent (Sigma-Aldrich). Total RNA (4 µg) was reverse-transcribed into cDNA using SuperScript III First-Strand Synthesis Kit (Invitrogen). PCR primers for *Amy1A* (F: TCCCTTTCGGTC CTCATCGTCC and R: CGCCTCGTTGCCGTA CTTAG) and the control gene *Actin* (F: AGGAATGGAAGCTGCGGGTAT and R: CAGGAGGACGGCGATAACA) were designed based on the coding sequences Os02g52710.1 and Os03g50885.1, respectively, available in the Gramene database (release 35 at http://www.gramene.org/Oryza_sativa). Real-time reactions were performed using the SYBER Green PCR Master Mix (Applied Biosystems Co). Primer validation and differential expression analysis were conducted using the method described in Chao (2008).

## Results

### Inheritance and QTL for plant height in the weed-cultivar cross

**Figure 2:**
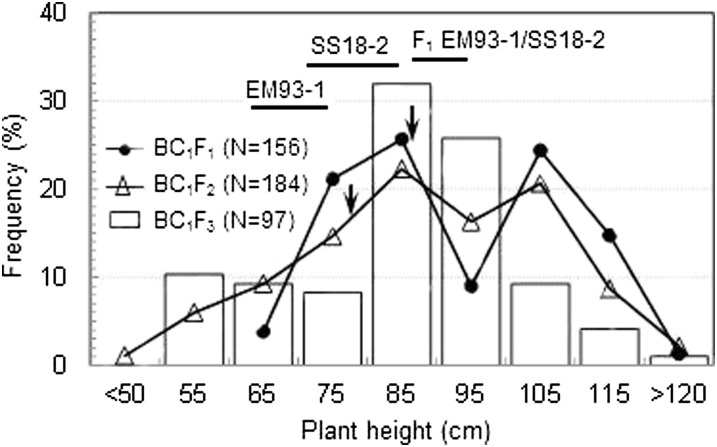
Frequency distributions of plant height in BC_1_F_1_, BC_1_F_2_, and BC_1_F_3_ populations. Horizontal bars represent the range of variation for the parental lines EM93-1 and SS18-2, or hybrid F_1_s. Arrows indicate the plant height for BC_1_F_1_ plant ^#^47 or BC_1_F_2_ plant ^#^101 selected to develop the BC_1_F_2_ or BC_1_F_3_ populations.

**Figure 3:**
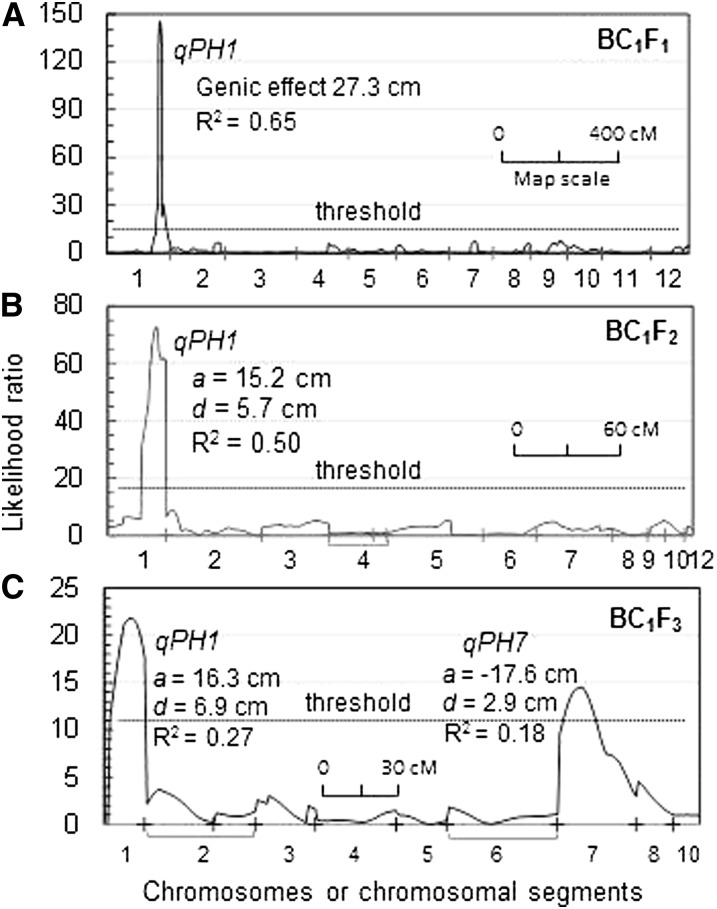
Scanning for plant height QTL segregating in the BC_1_F_1_ to BC_1_F_3_ populations (A−C). Horizontal axes are the total genetic distance in cM for chromosomes or chromosomal segments segregating in the populations (Figure 1B), with a bracket grouping two segments on one chromosome. QTL genic, additive (*a*), and dominance (*d*) effects and proportion of the variance (R^2^) were computed by the interval mapping procedure. A positive/negative effect indicates that the SS18-2-derived allele increasing/reducing plant height.

The data from the parental, F_1_ and BC_1_F_1_ generations revealed a complex inheritance pattern for plant height in the cross. Hybrid F_1_s (92 cm) were taller than the parents EM93-1 (73 cm) and SS18-2 (81 cm); the BC_1_F_1_s (60−126 cm) varied beyond the parental and F_1_ limits and displayed a bimodal distribution (Figure 2). A genome-wide scan detected only one QTL (*qPH1*) for plant height on chromosome 1, which accounted for 65% (R^2^) of the phenotypic variance in the BC_1_F_1_ population (Figure 3A). The major effect of *qPH1* could explain the bimodal distribution, but not the transgressive segregation. There must be undetectable QTL in the primary segregating population.

The BC_1_F_2_ was similar to the BC_1_F_1_ population for the bimodal distribution (Figure 2). QTL analysis confirmed *qPH1* (R^2^ = 50%) and detected no additional loci associated with plant height in the BC_1_F_2_ population (Figure 3B). The *qSD1-2* seed dormancy locus was detected in this BC_1_F_2_ population and colocated with *qPH1* in the RM315 to RM3602 region of ~2.3 Mb in physical length (Ye *et al.* 2010).

The BC_1_F_3_ was similar to the BC_1_F_2_ population for the range of variation in plant height (Figure 2). Two plant height QTL, *qPH1* and *qPH7*, were detected in the BC_1_F_3_ population (Figure 3C). The locus *qPH7* was located on chromosome 7 and accounted for less (R^2^ = 18%) phenotypic variance than *qPH1* (R^2^ = 27%). The *qPH1* allele from EM93-1 and the *qPH7* allele from SS18-2 reduced plant height. The distribution of height-reducing alleles across the two parents explained the transgressive segregation observed in the BC_1_F_1_, BC_1_F_2_, and BC_1_F_3_ populations. The *qSD7-2* seed dormancy QTL was detected in all three populations and co-located with *qPH7* in the RM346 to RM234 interval of ~6.3 Mb detected in BC_1_F_3_ population (Gu *et al.* 2004, Ye *et al.* 2010).

### Genetic effects of the isolated QTL clusters *qSD1-2/qPH1* and *qSD7-2/qPH7*

**Figure 4:**
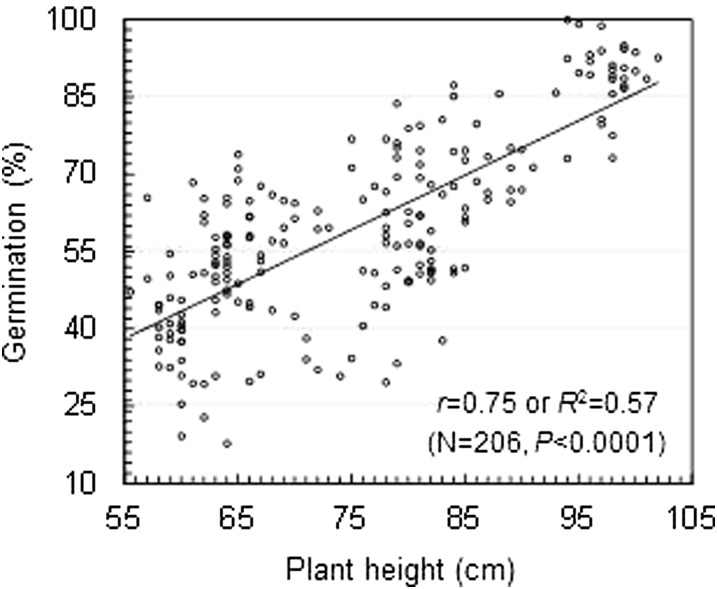
Scatter plot for plant height and seed dormancy. The data were collected from the field population segregating only for *qSD1-2/qPH1* and *qSD7-2/qPH7*. Seed dormancy was evaluated by percent germination of 14-d after-ripened seeds. Parameters shown are the number of plants (N), correlation (*r*) and determination (*R*^2^) coefficients, and probability level (*P*).

The two chromosomal segments introduced from SS18-2 cover the *qSD1-2/qPH1* and *qSD7-2/qPH7* peak-containing intervals, respectively. As expected, plant height correlated positively with germination rate in the digenic system consisting of the nine genotypes for the two segments (Figure 4). The linear correlation explained 48 and 57% of the phenotypic variances, respectively, in the greenhouse (*r* = 0.69) and field (*r* = 0.75) experiments. Genetic analysis demonstrated that both *qSD1-2/qPH1* and *qSD7-2/qPH7* had significant effects on both seed dormancy and plant height in the isogenic background (Table 1).

**Table 1:** Summary of genetic component effects of *qSD1-2/qPH1* and *qSD7-2/qPH7* on plant height and germination in greenhouse and field experiments

	Plant height, cm			Germination of 14-d after-ripened seeds		
Parameter*^a^*	Effect*^b^*	Prob.	R^2^ (%)*^c^*	Effect	Prob.	R^2^ (%)
Greenhouse experiment						
* µ* (intercept)	74.1	<0.0001	N/A	0.76	<0.0001	N/A
* a_1_*	16.4	<0.0001	82.2	0.14	<0.0001	30.2
* d_1_*	3.2	<0.0001	1.3	Not significant		
* a_2_*	−5.7	<0.0001	11.6	−0.16	<0.0001	40.8
* d_2_*	1.3	0.0045	0.2	Not significant		
* I_a1a2_*	−1.9	<0.0001	0.8	Not significant		
* I_d1a2_*	−1.6	0.003	0.2	Not significant		
Total (model R^2^)			96.2	71.0		
Field experiment						
* µ*	75.9	<0.0001	N/A	0.91	<0.0001	N/A
* a_1_*	13.1	<0.0001	81.4	0.13	<0.0001	31.1
* d_1_*	1.9	<0.0001	0.4	Not significant		
* a_2_*	−5.8	<0.0001	14.4	−0.15	<0.0001	34.6
* I_a1a2_*	−2.3	<0.0001	1.9	−0.07	<0.0001	5.7
Total (model R^2^)			98.0	71.3		

a Additive (*a*), dominance (*d*), and epistatic (*I*) effects were estimated based on Model 1, with the subscript 1 or 2 representing the *qSD1-2*/*qPH1* or *qSD7-2*/*qPH7* cluster.

b A positive (negative) value indicates that the parental line SS18-2 (EM93-1) contributed an effect-increasing allele to the cluster. The effect on germination is arcsine-transformed.

c R^2^, proportion of the variance explained by the component or model. N/A, not applicable.

Genetic component effects of the digenic system partitioned using Model 1 varied with the traits and environments (Table 1). For plant height, the phenotypic variation was attributed to additive (*a_1_* and *a_2_*) and some dominance (*d_1_* and *d_2_*) and epistatic (*I_a1a2_* and *I_d1a2_*) effects of *qPH1* and *qPH7* in the greenhouse, or to *a_1_*, *a_2_*, *d_1_*, and *I_a1a2_* in the field environment; *qPH1* had a greater main effect than *qPH7*. For seed dormancy, the phenotypic variation was attributed to additive effects (*a_1_* and *a_2_*) of *qSD1-2* and *qSD7-2* in the greenhouse, or to *a_1_*, *a_2_*, and their epistasis (*I_a1a2_*) in the field environment; *qSD7-2* had a greater additive effect than *qSD1-2*. Heritability was higher for plant height (0.96−0.98) than for seed dormancy (~0.71) across the two environments (Table 1).

**Figure 5:**
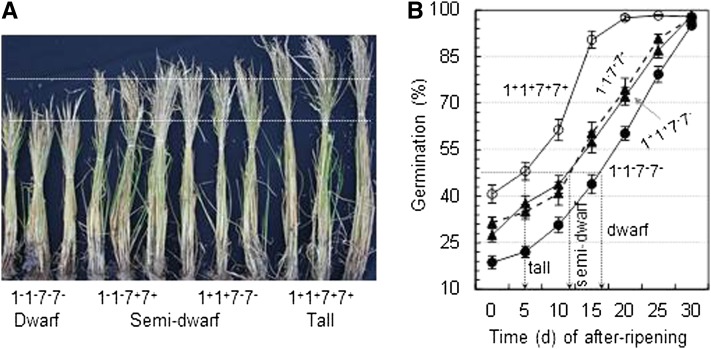
Genotypic differences in plant height and duration of seed dormancy. (A) Image of plants from four NILs. The NILs (1^−^1^−^7^−^7^−^, 1^−^1^−^7^+^7^+^, 1^+^1^+^7^−^7^−^, and 1^+^1^+^7^+^7^+^) fixed for effect-reducing (−) or -increasing (+) alleles at the chromosome-1 (*qSD1-2/qPH1*) or -7 (*qSD7-2/qPH7*) QTL, and represented three (dwarf, semidwarf, and tall) types of plant height. (B) Distributions of germination rates at a series of after-ripening time points. Data shown are means (circles) ± SE (bars) of 10 plants from a NIL for germination rates of seeds after-ripened for 0 to 30 d. Arrow-headed vertical lines point to the time period of after-ripening required for the genotype to reach 50% germination.

The four NILs showed three phenotypes for plant height (Figure 5A): dwarf (1^−^1^−^7^−^7^−^), semidwarf (1^+^1^+^7^−^7^−^ and 1^−^1^−^7^+^7^+^), and tall (1^+^1^+^7^+^7^+^). The two semidwarf genotypes were also similar in the duration of seed dormancy. It took about 16, 11, and 5 d of after-ripening for the dwarf, semidwarf, and tall genotypes to reach 50% germination, respectively, under the controlled conditions (Figure 5B). Therefore, the four digenic homozygotes displayed only three types of trait (plant height/seed dormancy) combinations: dwarf/strong dormancy, semidwarf/moderate dormancy, and tall/weak dormancy.

### Genetic dissection of *qSD1-2* from *OsVp1* and *qSD7-2* from *Sdr4*

**Figure 6:**
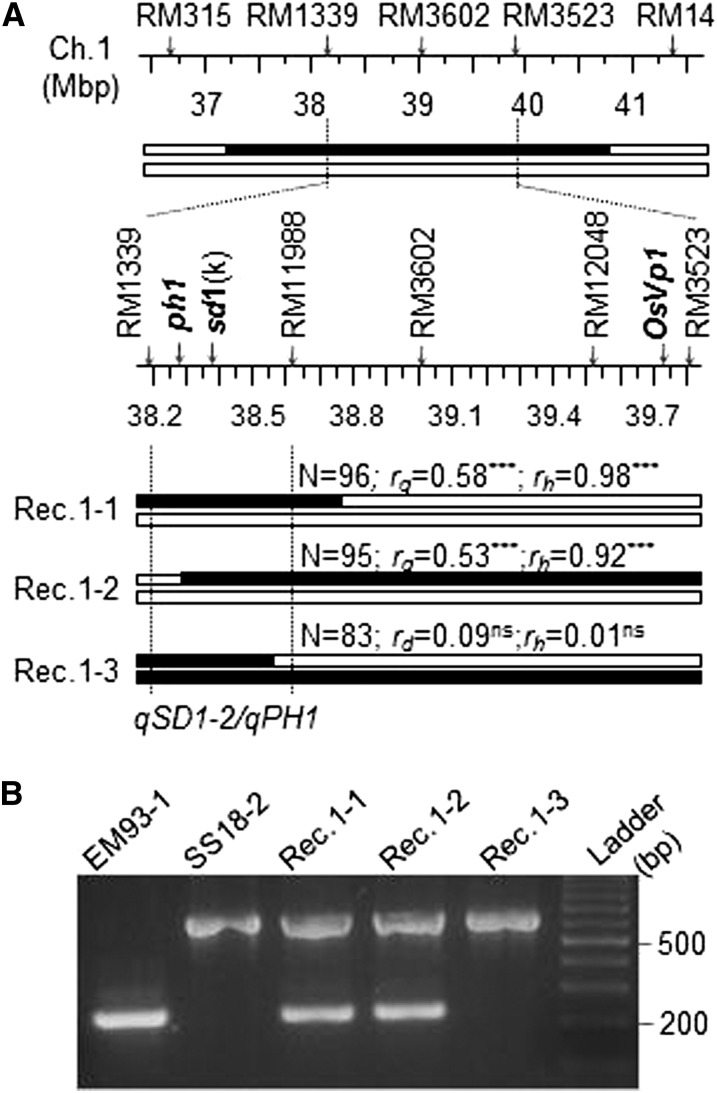
Fine mapping of the *qSD1-2/qPH1* region. (A) Physical map and recombinant genotypes. Markers on the partial map of chromosome 1 were aligned against the Nipponbare genome sequence (IRGSP 2005) and used to select recombinants (Rec.1-1 to -3) to dissect the initial introgression segment from SS18-2 (black bar in the top panel) in the EM93-1 background (empty bars). *OsVp1*, ortholog of the Maize *VIVIPAROUS1* gene; *ph1*, plant height QTL proposed to be *Os01g65900* (Kovi *et al.* 2011); and *sd1*(k), marker targeting the 383-bp functional deletion within the *sd1* semidwarf gene (Monna *et al.* 2002); N, the number of plants in a recombinant-derived progeny line; *r_g_* and *r_h_*, marker-trait correlation coefficients for germination (*r_g_*) or plant height (*r_h_*), with a significant value (*** for *P* < 0.0001 and ns for *P* = 0.05 or greater) indicating that the SS18-2-derived allele at locus or loci on the marked heterozygous region increased germination or plant height. Vertical lines delimit *qSD1-2/qPH1*, based on the progeny testing. (B) Gel image showing the polymorphism at *sd1*(k) among the parental and recombinant genotypes.

A partial high-resolution map for the *qSD1-2/qPH1* region located *sd1* and *OsVp1* on different marker intervals (Figure 6A). Three recombinants, which were heterozygous for *sd1* (Rec.1-1), both *sd1* and *OsVp1* (Rec.1-2), and *OsVP1* (Rec.1-3), respectively, were selected for progeny testing. Marker-trait correlations were significant only in the Rec.1-1 and -2 progeny lines, with *r* = 0.53−0.58 for germination and 0.92−0.98 for plant height. The absence of marker-trait association in the progeny line of Rec.1-3 suggests that *OsVp1* has no effect on germination in this background. The similar strength of correlation in the other two progeny lines suggests that *qSD1-2/qPH1* locates on the *sd1*-containing interval of <400 Kb between RM1339 and RM11988. Genetic analysis of the progeny data using Model 2 confirmed that the narrowed *qSD1-2/qPH1* cluster had an additive effect on germination and both additive and dominance effects on plant height, with the effect-increasing allele from SS18-2 (Supporting Information, Table S1).

Marker genotyping with *sd1*(k) demonstrated that the two parental lines differentiated at the site of the 383-bp functional deletion, with EM93-1 and SS18-2 carrying the mutant and wild-type alleles, respectively (Figure 6B). Both Rec.1-1 and -2 were heterozygous for *sd1*(k) and their progeny lines segregated for plant height (61-101 cm), whereas Rec.1-3 was an SS18-2-like homozygote at *sd1*(k) and its progeny line was true-breeding for the tall phenotype (100 cm) (Table S1). The consistence between marker genotypes and segregation patterns suggest that *sd1* is the underlying gene of *qPH1*.

**Figure 7:**
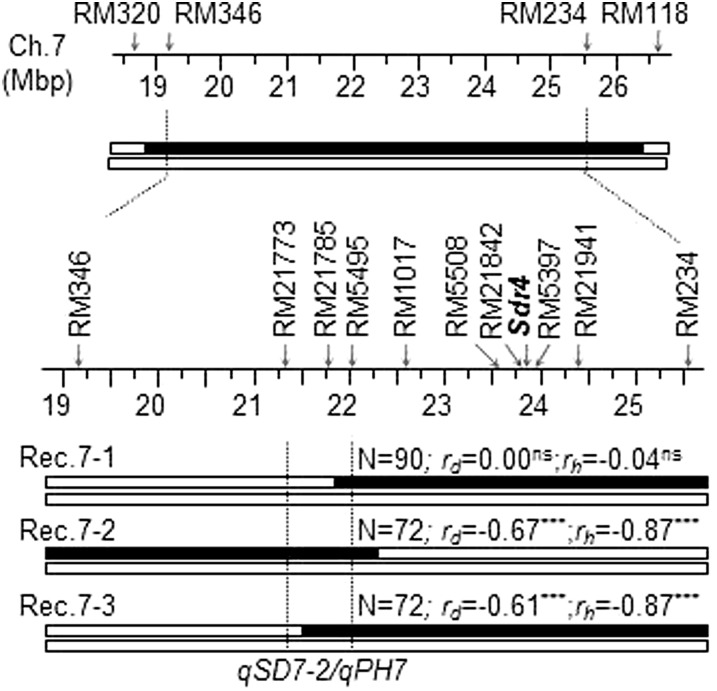
Physical map and selected recombinants for the *qSD7-2/qPH7* region. Markers on the partial map of chromosome 7 were aligned against the Nipponbare genome sequence (IRGSP 2005) and used to select recombinants (Rec. 7-1 to -3) to dissect the initial introgression segment from SS18-2 (black bar in the top panel) in the EM93-1 background (empty bars). *Sdr4*, seed dormancy gene; N, the number of plants in a recombinant-derived progeny line; *r_g_* and *r_h_*, marker-trait correlation coefficients for germination (*r_g_*) or plant height (*r_h_*), with a negative value indicating that the SS18-2-derived allele at a locus on the marked heterozygous region reduced germination or plant height; ***, significant at *P* < 0.0001; ns, not significant at *P* = 0.05 or greater. Vertical lines delimit *qSD7-2/qPH7*, based on the progeny testing.

A partial high-resolution map for the *qSD7-2/qPH7* region was developed with 10 polymorphic markers, including two flanking *Sdr4* (Figure 7). Three recombinants (Rec.7-1 to -3) were selected for progeny testing. Marker-trait correlations were not significant in the line from Rec.7-1, which was heterozygous for an *Sdr*4-containing segment, suggesting that there is no functional differentiation at *Sdr4* between the two parental lines. The correlations were significant in the two lines from Rec.7-2 and -3, which were heterozygous for an overlapping segment between RM21773 and RM5495, with *r* = 0.61−0.67 for germination and 0.87 for plant height, indicating that *qSD7-2/qPH7* locates on the RM21773 to RM5945 interval of <700 Kb. Genetic analysis of the progeny data using Model 2 confirmed that the narrowed *qSD7-2/qPH7* consisted of only additive effects, with the SS18-2-derived allele reducing germination and plant height (Table S1).

### Genotypic differences in GA response

**Figure 8:**
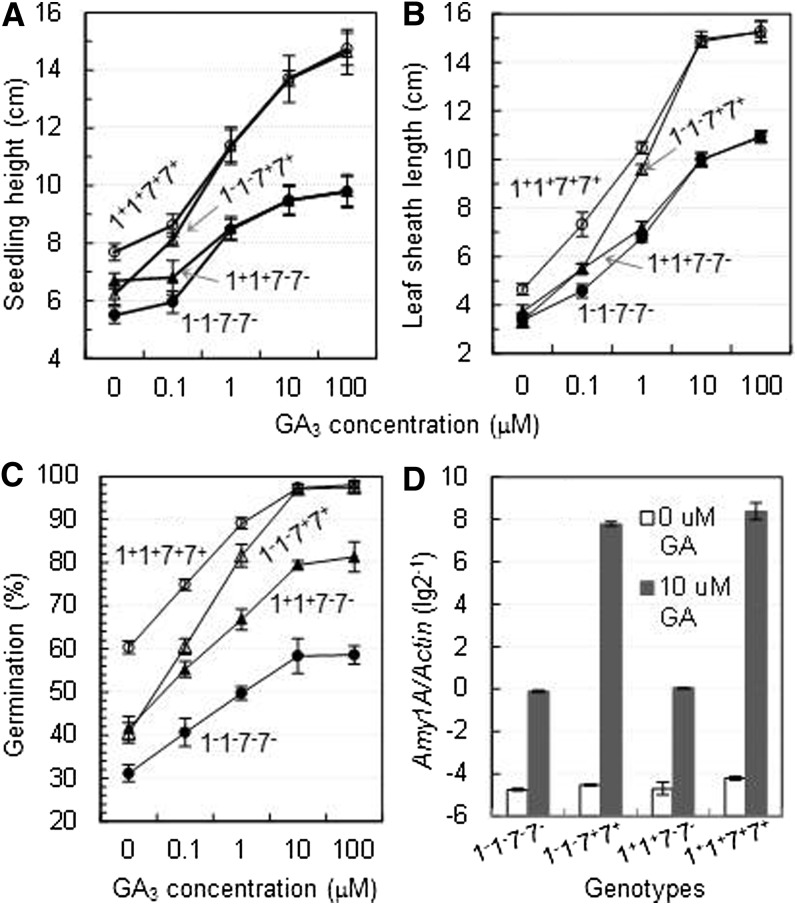
Genotypic differences of *qSD1-2/qPH1* and *qSD7-2/qPH7* in GA response. (A) Seedling height. (B) Leaf sheath length. (C) Germination. (D) Expression of the *α*-amylase 1A gene (*Amy1A*) in the endosperm tissue. Four near-isogenic lines are represented by combinations of alleles at chromosomes-1 (*qSD1-2/qPH1*) and -7 (*qSD7-2/qPH7*) QTL, with a superscript indicating the allele promoting (+) or reducing (−) both germination and seedling growth. Data shown are means (circles or triangles) and SDs (bars) of 10 plants for the (A) and (C), 18 plants for the (C), and 3 samples for the (D) experiments. Note that GA concentration units on the X axis for (A-C) are not uniformly scaled.

**Figure 9:**
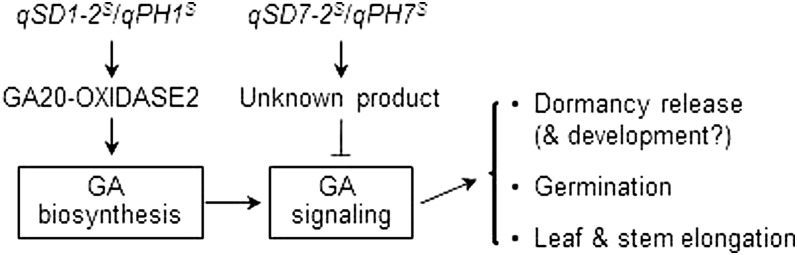
A working model to explain putative roles of *qSD1-2/qPH1 and qSD7-2/qPH7* in regulating seed dormancy, germination, and plant growth in rice. The letter S superscript to the QTL name indicates the allele from the weedy rice line SS18-2. GA20-OXIDASE2 is the enzyme required for GA biosynthesis in vegetative and seed tissues. The question marker indicates that these two naturally occurring genes may be also involved in regulation of seed dormancy development, as among the four digenic genotypes, the double mutant displayed the lowest response to exogenous GA applied during imbibition (Figure 8C).

The GA treatments generally promoted seedling elongation, germination, and the expression of *Amy1A* in the four NILs, but the promoting effects varied with the genotypes. For example, GA application completely recovered inhibiting effects on seedling height/leaf sheath length and germination for the *qSD1-2/qPH1* (1^−^1^−^7^+^7^+^ = 1^+^1^+^7^+^7^+^), but not for the *qSD7-2/qPH7* (1^+^1^+^7^−^7^−^ < 1^+^1^+^7^+^7^+^) single mutant (Figure 8, A−C). These data indicate that the natural variants of *qSD1-2/qPH1* and *qSD7-2/qPH7* belong to GA-sensitive and -insensitive mutants, respectively, and that *qSD1-2/qPH1* is likely involved in GA biosynthesis. The effect-reducing allele of *qSD7-2/qPH7* blocked the GA responses of the *qSD1-2/qPH1* wild type (*i.e.*, 1^+^1^+^7^−^7^−^ < 1^+^1^+^7^+^7^+^), including *Amy1A*’s transcription (Figure 8D), suggesting that *qSD7-2/qPH7* may work downstream of *qSD1-2/qPH1* to regulate plant height and seed dormancy (refer to Figure 9).

It was noticed that the genotypic differences of *qSD1-2/qPH1* in GA sensitivity varied between seedling height and seed dormancy. *qPH1*’s mutant effect on seedling elongation was compensated by ∼1 *μ*M GA (1^−^1^−^7^+^7^+^ = 1^+^1^+^7^+^7^+^ and 1^−^1^−^7^−^7^−^ = 1^+^1^+^7^−^7^−^), whereas *qSD1-2*’s mutant effect on germination was offset by ~10 *μ*M GA only when *qSD7-2* fixed for the effect-promoting allele (1^−^1^−^7^+^7^+^ = 1^+^1^+^7^+^7^+^; Figure 8, A−C). The difference in GA sensitivity indicates that cell elongation in the vegetative tissue is more sensitive to GA than germination, which is determined by the velocity of dormancy release during seed imbibition. In addition, the application of GA at 10 *μ*M or higher concentration could not compensate for the inhibiting effect of the 1^−^1^−^7^−^7^−^ genotype on germination (1^−^1^−^7^−^7^−^ < 1^+^1^+^7^−^7^−^ <1^−^1^−^7^+^7^+^ = 1^+^1^+^7^+^7^+^ in Figure 8C), suggesting that the QTL underlying genes may be also involved in the regulation of dormancy development.

## Discussion

### Consequences of the association between seed dormancy and plant height

This research established the association between seed dormancy and plant height in rice. The association is underlain by two clusters of QTL at which the alleles reducing germination also reduced stem elongation. The observed association appeared to help scatter the effect-reducing alleles in natural variants. For example, the *sd1* height-reducing allele was reported in two wild rice (*O. rufipogon*) accessions (Nagano *et al.* 2005), and the *qSD7-2/qPH7* mutant allele is present in the weedy rice accession SS18-2. This scattering distribution was likely because a dwarf phenotype, which is caused by an accumulation of the height-reducing alleles from different loci, has no selective advantage in natural populations. Of the 10 validated seed dormancy QTL differentiated between EM93-1 and SS18-2 (Ye *et al.* 2010), two (or 20%) were colocalized with the QTL for plant height. This finding suggests that a selection for/against seed dormancy would have about 20% chance to retain/eliminate a height-reducing allele. Therefore, the seed dormancy-plant height association represents a balancing mechanism that helps maintain the genetic diversity for these two adaptive traits in natural populations.

One more beneficial effect of the “Green Revolution” gene *sd1* that has not been described would be the association with an increase in seed dormancy, due to a tight linkage or pleiotropy. The increased effect lengthened the after-ripening time by ~5 d to reach 50% germination (Figure 5). This effect must have contributed to the resistance of semidwarf cultivars to preharvest sprouting in rice production in the past decades. The *qSD7-2/qPH7* allele isolated from SS18-2 is similar to *sd1* and could be used as an alternative gene to breed semidwarf varieties with an enhanced resistance to preharvest sprouting.

The reduction in plant height for major crops after the “Green Revolution” imposed a selection force on accompanying weeds. Morphological mimicry is an evolutionary mechanism regulating weed adaptation to agro-ecosystems to avoid eradication by agricultural practices (Barrett 1983; Smith 1988). This hybridization experiment identified genetic bases underlying the weed mimicry for semidwarf plant height. A semidwarf genotype could be defined as nonallelic combinations of at least two loci, with one fixed for an effect-increasing and the other fixed for an effect-reducing allele (Figure 5A). The genetic diversity for the associated traits, or only for plant height, maintained in wild and weed populations could contribute to the morphological mimicry. The semidwarf weed line SS18-2 is such an example, as the *qPH7* allele from the parent offsets the effect of the *qPH1* wild-type allele on plant height in the weed genotype. For conspecific weeds, the introgression of a semidwarf gene from accompanying cultivars by outcrossing, or “gene flow,” could also contribute to the mimicry. The development of two morphologically identical semidwarf NILs (Figure 5) would be a simulation of the introgression. Recent research detected an *sd1*-like haplotype in three of 58 weedy rice accessions, which was presumably derived from the gene flow from the local semidwarf cultivars (Reagon *et al.* 2011).

### Shared mechanisms regulating the associated traits

This research isolated GA-sensitive (*qSD1-2/qPH1*) and GA-insensitive (*qSD7-2/qPH7*) natural mutants and provided a set of isogenic materials to test a model for shared mechanisms regulating both seed dormancy and plant height (Figure 9). In addition to seedling and plant height, *qSD1-2/qPH1* was also associated with the duration of seed dormancy, the functional 383-bp deletion mutation in *OsGA20ox2*, the change in expression of the GA-inducible gene *Amy1A*, and GA sensitivity for germination (Figure 8). This series of associations strongly suggest that *OsGA20ox2* is a *qSD1-2/qPH1*-underlying gene, which controls plant height and likely also affects seed dormancy and germination by regulating GA biosynthesis in developing and/or imbibed seeds. The GA level was reduced in stems of the *OsGA20ox2* mutants, which accounted for the semidwarf phenotype (Ashikari *et al.* 2002; Monna *et al.* 2002; Spielmeyer *et al.* 2002; Kaneko *et al.* 2003). However, differences between the *OsGA20ox2* wild-type and mutant genotypes for the GA level in developing and imbibed seeds and for the degree of seed dormancy were not determined, which could have contributed to the uncertainty about the role of GA in regulating germination in rice (Steber 2007). The plant height QTL *ph1* was mapped onto the *qSD1-2/qPH1* region of <400 kb and proposed to be the locus *Os01g65900* encoding a predicted chitin-inducible GA-responsive protein (Kovi *et al.* 2011). Research is being conducted to characterize the regulatory role of *OsGA20ox2* in dormancy development and release and to determine if *Os01g65900* also contributes an effect to *qPH1*,or *qSD1-2*, or both.

The genetic analysis based of the double mutants provided evidence that *qSD7-2/qPH7* works downstream of *qSD1-2/qPH1* or the GA signaling pathway (Figure 9). In the narrowed *qSD7-2/qPH7* region of <700 Kb, there is no gene or predicted paralog with a known function for GA biosynthesis or signaling. It is possible that *qSD7-2/qPH7* is a new gene controlling the associated traits through GA signaling. Research is also being conducted to clone and characterize *qSD7-2/qPH7*.

## Supplementary Material

Supporting Information
