# Supplementary material for: Genetic and Physiological Characterization of Two Clusters of Quantitative Trait Loci Associated With Seed Dormancy and Plant Height in Rice
Source: G3 (Bethesda). 2013 Feb 1;3(2):323–31. doi: 10.1534/g3.112.005041 (PMC3564992; doi:10.1534/g3.112.005041)
Supplement: Supporting Information [file supp_3_2_323__index.html]

Supporting Information 

# Genetic and Physiological Characterization of Two Clusters of Quantitative Trait Loci Associated With Seed Dormancy and Plant Height in Rice

## Supporting Information for Ye *et al.*, 2013

**Files in this Data Supplement:**

- Supporting Information - File S1 and Table S1 (PDF, 155 KB)
- Table S1 - Summary of marker-assisted progeny testing for six recombinants on the *qSD1-2/qPH1* or *qSD7-2/qPH7* region (PDF, 101 KB)
- File S1 - Raw data and result .csv files (.zip, 83 KB)
