# Supplementary material for: Genetic and Physiological Characterization of Two Clusters of Quantitative Trait Loci Associated With Seed Dormancy and Plant Height in Rice
Source: G3 (Bethesda). 2013 Feb 1;3(2):323–31. doi: 10.1534/g3.112.005041 (PMC3564992; doi:10.1534/g3.112.005041)
Supplement: Supporting Information [file supp_3.2.323_TableS1.pdf]

Table S1 Summary of marker-assisted progeny testing for six recombinants on the *qSD1-2/qPH1* or *qSD7-2/qPH7* region. Markers that were heterozygous in the recombinants were evaluated for genotypic means and gene additive (*a*) and dominance (*d*) effects on germination and plant height in the progeny lines.

QTL region	Recom-binant[a]	Progeny line[b]							
		Geno-type	N	Germination (%) of 7-d after-ripened seeds			Plant height (cm)		
				Mean ± SD	*a* (Prob.)	*d* (Prob.)	Mean ± SD	*a* (Prob.)	*d* (Prob.)
qSD1-2 /qPH1	Rec.1-1 (RM11988)	EE	25	30.2 ± 14.2	11.0 (<0.0001)	-0.4 (0.86)	61.3 ± 2.2	19.8 (<0.0001)	1.5 (0.008)
		ES	48	40.8 ± 11.2			82.7 ± 2.6		
		SS	23	52.1 ± 8.3			101.0 ± 3.5		
	Rec.1-2 (RM11988)	EE	19	37.9 ± 11.9	12.4 (<0.0001)	0.2 (0.96)	63.9 ± 2.6	17.9 (<0.0001)	1.2 (0.06)
		ES	54	50.5 ± 10.8			83.1 ± 3.4		
		SS	22	62.7 ± 18.5			99.7 ± 2.7		
	Rec.1-3 (RM3602)	EE	21	51.5 ± 12.0	(Fixed for SS18-2 allele at *qSD1-2*)		100.6 ± 4.4	(Fixed for SS18-2 allele at *qPH1*)	
		ES	42	50.9 ± 9.7			100.7 ± 2.9		
		SS	20	51.5 ± 5.7			100.8 ± 3.8		
qSD7-2 /qPH7	Rec.7-1 (RM21842)	EE	30	50.4 ± 14.1	(Fixed for EM93-1 allele at *qSD7-2*)		61.2 ± 1.8	(Fixed for EM93-1 allele at *qPH7*)	
		ES	30	48.8 ± 10.7			60.3 ± 2.4		
		SS	30	50.4 ± 11.8			61.0 ± 2.3		
	Rec.7-2 (RM346)	EE	18	35.4 ± 6.9	-12.2 (<0.0001)	-2.2 (0.28)	61.0 ± 1.5	-3.2 (<0.0001)	-0.3 (0.30)
		ES	37	20.8 ± 12.1			57.4 ± 1.3		
		SS	17	11.1 ± 3.3			54.5 ± 1.3		
	Rec.7-3 (RM21842)	EE	20	33.0 ± 10.7	-10.0 (<0.0001)	-1.0 (0.68)	60.5 ± 1.9	-4.0 (<0.0001)	-0.3 (0.47)
		ES	35	22.0 ± 11.0			56.1 ± 1.6		
		SS	17	13.0 ± 8.9			52.4 ± 1.7		

[a] Refer to Figures 6A and 7 for genotypes of markers listed in the parentheses.

[b] Letter E or S indicate the parental origin of the allele from EM93-1 or SS18-2. A positive (or negative) additive (*a*) effect indicates that the S (or E) allele promoting germination or stem elongation.
